# Breast Cancer Spheroids Reveal a Differential Cancer Stem Cell Response to Chemotherapeutic Treatment

**DOI:** 10.1038/s41598-017-10863-4

**Published:** 2017-09-04

**Authors:** Daniel S. Reynolds, Kristie M. Tevis, William A. Blessing, Yolonda L. Colson, Muhammad H. Zaman, Mark W. Grinstaff

**Affiliations:** 1 Department of Biomedical Engineering, Boston University, Boston, MA 02215 USA; 2 Division of Thoracic Surgery, Department of Surgery, Brigham and Women’s Hospital, Boston, MA 02215 USA; 3 Department of Chemistry, Boston University, Boston, MA 02215 USA; 4 Howard Hughes Medical Institute, Boston University, Boston, MA 02118 USA; 5 Department of Medicine, Boston University School of Medicine, Boston, MA 02118 USA

## Abstract

An abnormal multicellular architecture is a defining characteristic of breast cancer and, yet, most *in vitro* tumor models fail to recapitulate this architecture or accurately predict *in vivo* cellular responses to therapeutics. The efficacy of two front-line chemotherapeutic agents (paclitaxel and cisplatin) are described within three distinct *in vitro* models employing the triple-negative basal breast cancer cell line MDA-MB-231 and the luminal breast cancer cell line MCF7: a) a 3D collagen embedded multicellular spheroid tumor model, which reflects the architecture and cellular heterogeneity of tumors *in vivo*; b) a 3D collagen model with a single cell-type diffusely embedded; and c) a 2D monolayer. The MDA-MB-231 embedded spheroid tumor model exhibited the most robust response to chemotherapeutic treatment, and possessed the greatest cancer stem cell (CSC) content. CSC-related genes are elevated across all MDA-MB-231 *in vitro* models following paclitaxel treatment, indicating that paclitaxel enrichment of chemoresistant CSCs is less dependent on microenvironmental tumor structure, while cisplatin showed a more context-dependent response. In the MCF7 cell models a context-dependent response is observed with paclitaxel treatment increasing the CSC related genes in the 2D monolayer and 3D diffuse models while cisplatin treatment afforded an increase in *ALDH1A3* expression in all three models.

## Introduction

One of the defining characteristics of solid tumors is an abnormal multicellular architecture^1^. This aberrant architecture is known to further drive tumor progression through forced-depolarization, enhanced cell-cell contacts, and loss of tensional homeostasis^2^. Moreover, it leads to the formation of a heterogeneous environment characterized by gradients of metabolites, catabolites, and oxygenation, which form around leaky, tortuous vasculature^3^. As a result, a range of metabolic states exists in which cells adjacent to capillaries are well–perfused, metabolically active, and experience significant cell-ECM contacts. This is in contrast to cells at the tumor center that are often either quiescent or apoptotic, exposed to hypoxia and low pH, and exhibit increased cell-cell contacts^4, 5^. Tumor heterogeneity is not limited to spatial differences in metabolic activity, as it also includes cellular phenotypic heterogeneity with the presence of a subpopulation of highly malignant cancer cells, known as cancer stem cells (CSCs), which are linked to chemoresistance, metastasis, and progression *in vivo* ^6, 7^. The high malignancy potential of CSCs arises from their ability to both self-renew and form more-differentiated progeny that compose the bulk of the tumor cell population^8^. Despite the evidence showing that abnormal multicellular architecture and CSCs contribute to tumor progression, the impact of multicellular architecture on CSC content is not well understood due to the lack of robust, scalable, and quantitative *in vitro* models.

Attempts to accurately recapitulate the *in vivo* cellular response to therapeutic assault, especially that of apparent quiescent CSCs, is hindered by the absence of robust microenvironmental models that recreate *in vivo* characteristics and cellular relationships. The need for improved pre-clinical models is recognized at the populous level in the US with the announcement of the Precision Medicine Initiative^9^. Traditionally, cells investigated for form, function, and/or response to molecular or macromolecular agents, are typically cultured on artificial 2D polystyrene or other planar systems. This planar 2D growth geometrically constrains the cells, forcing an artificially imposed basal lateral attachment, resulting in genetic upregulation of cell cycling and metabolism as manifested through enhanced proliferation and extreme cell spreading^10, 11^. To improve upon the limitations inherent in 2D systems, the current study utilizes 3D multicellular spheroids to mimic important relational characteristics observed within tumors *in vivo*. These multicellular spheroids, which are hundreds of micrometers in diameter, possess cell-cell contacts, a necrotic core, and gradients of proliferation^12, 13^. Furthermore, by embedding within 3D collagen, multicellular spheroids reside within an extracellular matrix (ECM) similar to that found *in vivo*, providing additional signaling and mechanical cues^3^. Thus, the embedded multicellular spheroid model described herein more closely recapitulates the native 3D tumor microenvironment than conventional 2D culture, facilitating the study of tumor cell behavior within a more physiologically relevant context.

Use of the embedded spheroid model enables drug efficacy to be studied as a function of multicellular architecture. Results from published cell cytotoxicity assays using embedded spheroids, as compared to 2D monolayers or non-embedded spheroids, demonstrate increased cell robustness towards the chemotherapeutic agent when spheroids are embedded^14–16^. However the origin of this robust chemoresistive response and the effects of collagen embedment (i.e., matrix effects) are not well characterized. Therefore, we report the efficacy and treatment response of two front-line chemotherapeutics (paclitaxel and cisplatin) against the triple-negative basal breast cancer cell line, MDA-MB-231, and the luminal breast cancer cell line, MCF7, plated in a standard 2D monolayer vs. embedded within 3D collagen gels either as diffusely embedded single cells (termed ‘3D diffuse’) or as multicellular spheroids (termed ‘3D embedded spheroid’). Because selective enrichment and failure to completely eradicate the highly malignant CSC subpopulation within a tumor is thought to be a significant driver of cancer relapse, particularly following cytotoxic chemotherapy^6^, we investigated CSC content following treatment with the aforementioned chemotherapies as a function of these *in vitro* models. Specifically, we report: 1) collagen embedded models of diffuse and spheroid breast cancer cells are feasible, robust, reliable, and architecturally distinct from one another; 2) drug response (metabolic and growth prevention) differs between tumor cells located in the spheroid core and the spheroid periphery; 3) drug response and CSC content is dependent on breast cancer cell type used in the three models; 4) CSC content is greater and spatially-dependent within the spheroid; and 5) tumor architecture and the related microenvironmental cues influence the differential response of CSC enrichment following treatment with chemotherapy.

## Results

### Qualitative Model Response to Drug Treatment

**Figure 1:**
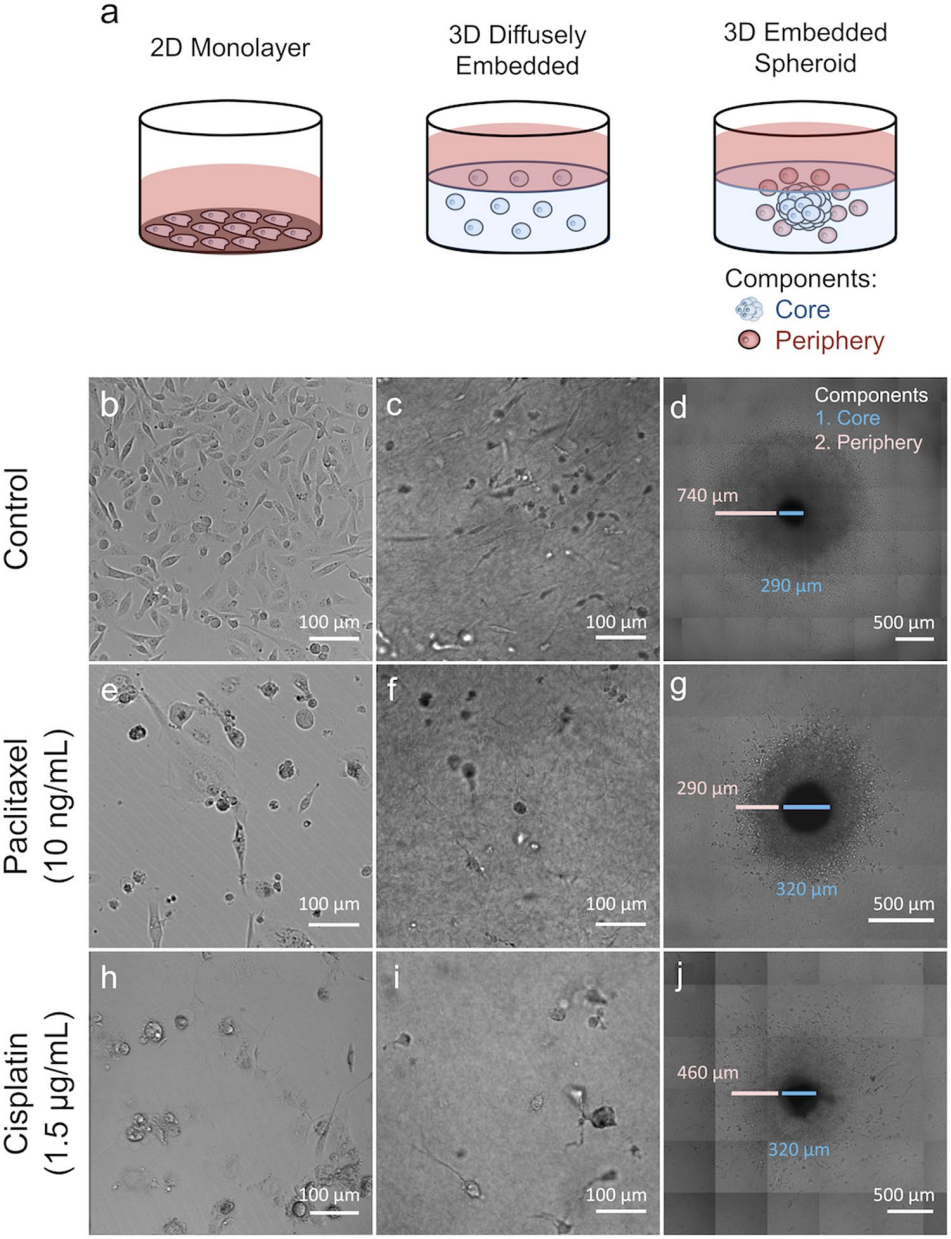
*In vitro* MDA-MB-231 tumor models respond to paclitaxel and cisplatin. The culture models used were a 2D monolayer, and two 3D models (single-cells diffusely embedded in a collagen gel or a collagen embedded multicellular spheroid) (**a**). The 3D spheroid has two distinct cell populations, a compact core and a periphery composed of cells in the surrounding matrix. DIC imaging shows the 2D monolayer untreated (**b**), after treatment with paclitaxel (10 ng/mL) (**e**), and after treatment with cisplatin (1.5 μg/mL) (**h**) on the eighth day of culture. The 3D diffusely embedded model is similarly shown untreated (**c**), after treatment with paclitaxel (**f**), and after treatment with cisplatin (**i**) on the eighth day of culture. The 3D embedded spheroid is shown untreated (**d**), after treatment with paclitaxel (**g**), and after treatment with cisplatin (**j**) on the eighth day of culture. The relative sizes of the core (blue) and periphery (pink) are marked.

The effect of three-dimensional culture and tumor macrostructure on tumor drug response was explored by comparing three different cell culture models using the post-metastatic, triple-negative breast cancer cell line MDA-MB-231 (Fig. 1) and the luminal breast cancer cell line, MCF7. The first model, a 2D monolayer, is the conventional method traditionally utilized for assaying drug efficacy *in vitro* (Fig. 1b). The “3D diffuse” model recreates a three-dimensional microenvironment by diffusely seeding a single cell suspension within a 4 mg/mL collagen gel (Fig. 1c), but the diffuse nature fails to recapitulate the enhanced cell-cell interactions characteristic of intact tumors *in vivo*. The third model, a “3D embedded spheroid” model, contains a multicellular spheroid of approximately 10^4^ cells (433 ± 34 μm and 541 ± 80 µm in diameter for MDA-MB-231 and MCF7 spheroids, respectively) embedded within a 4 mg/mL collagen gel (Fig. 1d); thus incorporating tumor macrostructure, cell-cell contacts, and the three-dimensional tumor architecture. In this model, the spheroids are first formed via liquid overlay and then transferred to collagen, following a method established in our previous publication^17^. In our 3D embedded spheroid model, we define cells that have invaded away from the spheroid and into the surrounding collagen as belonging to the “spheroid periphery” subpopulation, and define cells remaining within the spheroid as belonging to the “spheroid core” subpopulation, which appear as a black circle in DIC images (Fig. 1d).

In the 2D monolayer and 3D diffuse culture models of the MDA-MB-231 cells, an increase in rounded, spherical cells was observed after treatment with either paclitaxel (Fig. 1e,f), which prevents microtubule disassembly during cell division^18, 19^, or cisplatin (Fig. 1h,i), which crosslinks DNA^20, 21^. This rounded morphology following treatment is in contrast to the elongated morphology observed with untreated cells (Fig. 1b,c).

The observed cellular response to treatment within the 3D embedded MDA-MB-231 spheroid model was markedly different, manifesting response through changes in tumor cell growth and invasion into the surrounding matrix rather than the rounded morphology of individual tumor cells noted above for the other cell culture models. When treated with paclitaxel, spheroid ingrowth into the collagen matrix, i.e., the ‘periphery’ of the spheroid, was significantly decreased resulting in a small, dense structure measuring 291 ± 5 μm compared to 842 ± 8 μm (p < 0.01) in untreated controls (Fig. 1g). Similar treatment with cisplatin also decreased cell ingrowth into the collagen, albeit to a lesser extent (474 ± 15 μm, when compared to untreated controls, p < 0.01) (Fig. 1j), with visible cells migrating far into the collagen similar to the untreated control. In contrast to the changes noted in the ‘periphery,’ no morphological differences were observed within the dense spheroid ‘core’, which measured 304 ± 28 μm in DIC images (Fig. 1d,g,j), regardless of treatment. Although invasion is a qualitative measurement in this context, there is an observable increased sensitivity of the spheroid periphery cells to both chemotherapeutics compared to the apparent tumor resiliency present within the spheroid core. To confirm that these differences are not due to differences in drug exposure and that drug can penetrate throughout the spheroid, a fluorescent analog of paclitaxel was exposed to the spheroid for 72 hours. Confocal microscopy of the paclitaxel-treated spheroid demonstrated drug presence throughout the cells of the spheroid including penetration into the spheroid core (Supplementary Fig. 1). Using a computational diffusion model of our collagen gels, based on measured diffusion rates, the concentrations of paclitaxel and cisplatin reached equilibrium within the first 12 hours (Supplementary Fig. 1).

Given these qualitative morphological differences, we next investigated the effect of each chemotherapeutic agent against the various tumor culture models using quantitative metrics of cell viability, growth, oxygen consumption, and stem cell presence.

### Cell Viability after Drug Treatment

**Figure 2:**
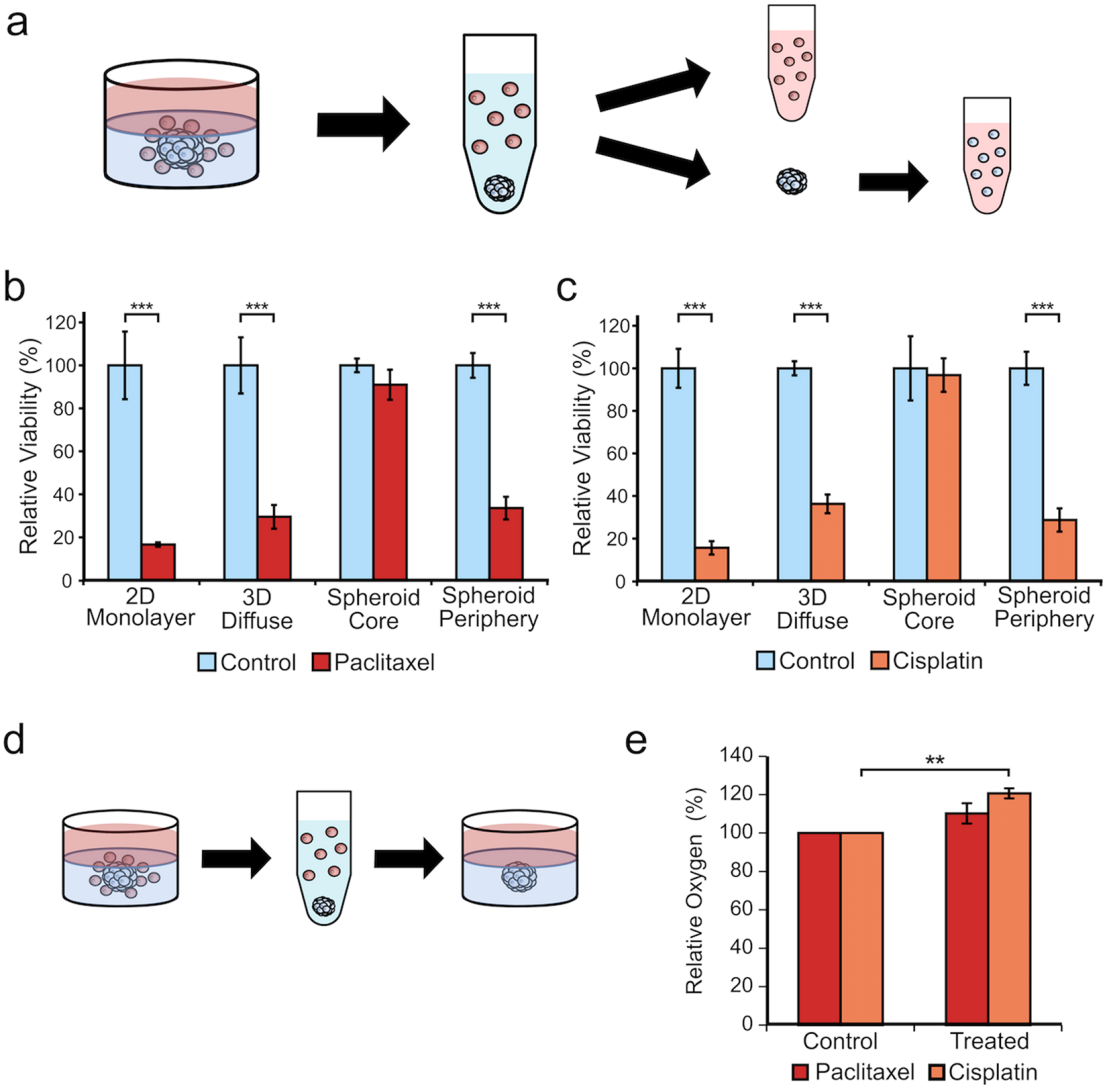
Quantitative assessment reveals resilience of spheroid core to chemotherapeutic treatment. The core and periphery populations of the 3D embedded MDA-MB-231 spheroid model can be separated by subsequent disaggregation in collagenase for the periphery and trypsin for the core (**a**). Viability assessment of both spheroid populations alongside the two other culture models following treatment with paclitaxel (**b**) or cisplatin (**c**). Data presented as mean ± SD; n = 6. P value determined using Student’s t-test (***P < 0.001). Representative set of three independent experiments shown. In order to assess the ability of the core cells to grow after treatment, the spheroid was treated with paclitaxel or cisplatin, the core was then removed and embedded into a new collagen gel, and cultured (**d**). After growth in the new collagen gel, the oxygen presence of treated spheroid cores was tested as a metric of growth (**e**). Data presented as mean ± SD; n = 3. P value determined using Student’s t-test (**P < 0.01).

Tumor cell viability for each MDA-MB-231 model was quantified before and after incubation with the individual chemotherapeutic agents using a colorimetric metabolic assay (Fig. 2b,c). Cells growing in the 2D monolayers were the most sensitive to chemotherapeutic intervention and exhibited viabilities of only 17% and 16% after treatment with paclitaxel and cisplatin, respectively. To assess tumor viability in the 3D diffuse model, the MDA-MB-231 cells were isolated from the surrounding collagen by a short treatment with collagenase prior to being re-seeded in a 2D monolayer culture for 12 hours so that our standard tumor cytotoxicity assay could be performed in the same fashion for all cells. Paclitaxel and cisplatin treatment decreased tumor cell viability to 30% and 36%, respectively, compared to untreated controls. For the 3D embedded MDA-MB-231 spheroid model, two cell populations were isolated and studied: the solid core and the peripheral spheroid cells, which are diffusely arranged around the core as shown in Fig. 2a. Collagenase was first used to disaggregate the collagen away from the spheroid, leaving a suspension of peripheral cells and the intact core. The core was then isolated and subsequently disaggregated with trypsin allowing the two separate cell populations (periphery and core) to be separately assayed. We recognize that this procedure is a limitation of our study as opposed to assessing viability of the periphery and core cells directly in the embedded spheroid. Even though the peripheral cells responded to paclitaxel and cisplatin akin to cells in the diffuse 3D model, with viabilities of 34% and 29%, respectively, the tumor cells residing in the core were markedly more resistant with viabilities of 91% and 97% for paclitaxel and cisplatin, respectively.

### Growth After Treatment

Given the marked disparity in tumor cell viability noted following chemotherapy exposure between those MDA-MB-231 tumor cells that reside in the periphery and those within the core, we quantitatively assessed the proliferative capability of the chemotherapy-treated core cells as a means to better understand the relationship of resilient growth as a function of tumor architecture and location. Following drug treatment, the core was isolated and embedded into a new collagen gel (4 mg/mL), and cultured for 8 days, as illustrated in Fig. 2d. Overall metabolic activity and growth were monitored via imaging (Supplementary Fig. 2) and oxygen consumption (Fig. 2e). MDA-MB-231 spheroids previously treated with paclitaxel or cisplatin exhibited growth as monitored by DIC imaging. With regards to oxygen consumption, the paclitaxel treated cells afforded a slight, but not significant, increase in oxygen presence when compared to the untreated control, suggesting that paclitaxel does not prevent subsequent growth (Fig. 2e). Spheroids treated with cisplatin exhibited significantly higher oxygen presence (p < 0.01), indicating that cisplatin is more effective at retarding proliferation, but does not completely inhibit it.

### Quantifying Cancer Stem Cells as a Function of Tumor Model

Among the three *in vitro* tumor models, MDA-MB-231 cells from within the “3D embedded tumor spheroid” following chemotherapy exposure demonstrated preserved cellular morphology, slow but continued growth, and markedly better cell viability, especially within the central spheroid core. These findings suggest that placing tumor cells within the more relevant 3D tumor architecture results in a more resilient, and clinically relevant, tumor cell phenotype. Given that cancer stem cells (CSC) have been shown to exhibit significant chemotherapy resistance *in vivo* and can be increased in frequency following chemotherapy^22–25^, we assessed CSC content across the three *in vitro* models in order to determine whether the differences identified in drug efficacy correlated with an enriched CSC content. First, we determined the CSC content within each of the untreated *in vitro* MDA-MB-231 models using three separate techniques: (1) ALDEFLUOR assay, which identifies CSCs according to their high aldehyde dehydrogenase (ALDH) enzymatic activity via a fluorescent substrate^26^; (2) Mammosphere assay whereby CSCs’ self-renewal properties are exploited to form clusters in non-adherent conditions^27^; and (3) RT-qPCR for *ALDH1A3, SOX2*, *OCT4*, and *NANOG*, known genetic markers for CSCs^28–32^.

**Figure 3:**
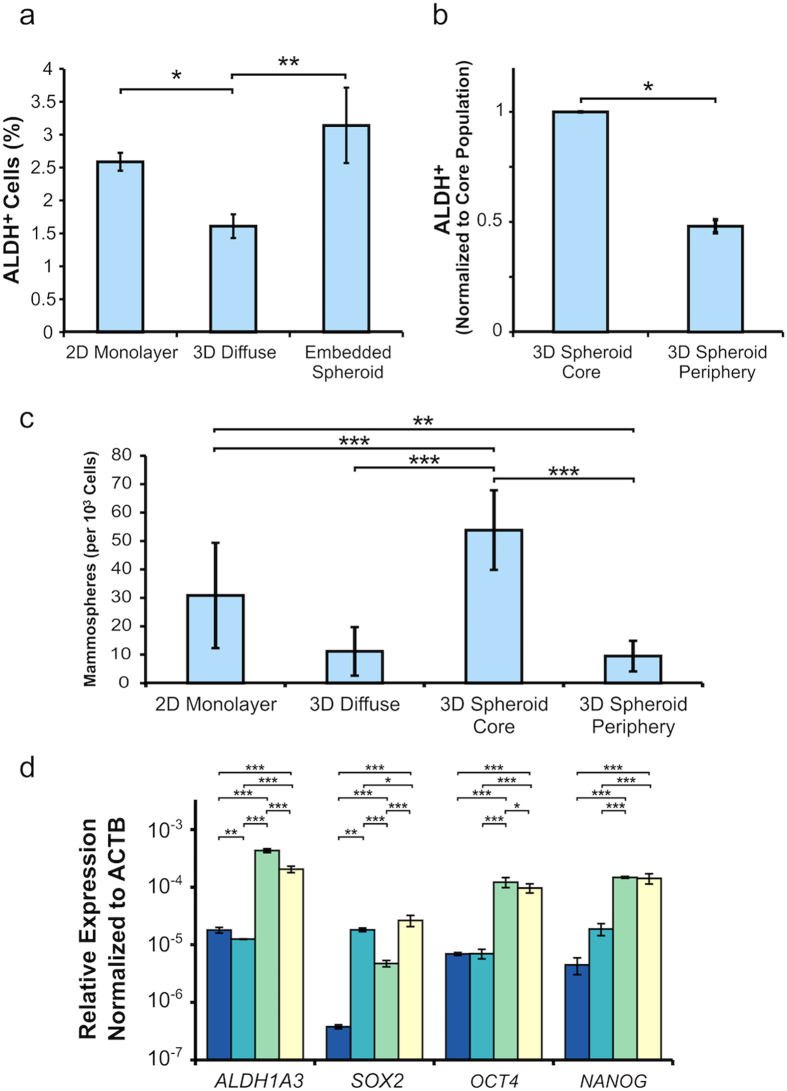
CSC markers are enriched within the 3D embedded core population of MDA-MB-231 spheroids. (**a**) ALDEFLUOR assay results from the three untreated *in vitro* tumor models. Error bars represent mean ± SD; n = 3. *P* value was determined using ANOVA with Tukey-Kramer post hoc analysis (*P < 0.05, **P < 0.01). (**b**) ALDEFLUOR assay results for untreated 3D spheroid core and periphery populations (normalized to core population). Error bars represent mean ± SD; n = 3. *P* value was determined using Student’s t-test (*P < 0.05). (**c**) Mammosphere assay results from the untreated *in vitro* models. Error bars represent mean ± SD; n = 3. *P* value was determined using ANOVA with Tukey-Kramer post hoc analysis (**P < 0.01, ***P < 0.001). (**d**) RT-qPCR analysis of *ALDH1A3*, *SOX2, OCT4*, and *NANOG* gene expression in the untreated *in vitro* models. Gene expression is normalized to *ACTB* expression as an internal control. Error bars represent mean ± SD; n = 3. *P* value was determined using ANOVA with Tukey-Kramer post hoc analysis (**P < 0.01, ***P < 0.001).

The ALDEFLUOR assay revealed that the 3D diffuse model exhibited significantly fewer ALDH + cells than either the 2D monolayer or the 3D embedded spheroid models (Fig. 3a). Isolation and characterization of the core versus the periphery populations in the 3D embedded spheroids demonstrated that CSC ALDH + cells were preferentially located in the core at an approximate ratio of 2:1 (Fig. 3b). These results were independently corroborated by the mammosphere assay showing a similar trend with a ratio of CSCs in the core versus the periphery being slightly above 5:1 (Fig. 3c).

The findings were also confirmed via RT-qPCR analysis with *ALDH1A3* expression reflecting the ALDEFLUOR results presented above. In particular, compared to the untreated 2D monolayer, *ALDH1A3* and thus CSC content was slightly downregulated (0.70-fold; p < 0.01) in the 3D diffuse culture. In contrast, the *ALDH1A3* CSC marker is markedly elevated in both the 3D spheroid core (24-fold; p < 0.001) and 3D spheroid periphery (11-fold; p < 0.001), as shown in Fig. 3d, suggesting that the 3D architecture and resultant cell-cell interactions that occur within tumor spheroids also translates to an increase in CSC content and presumably increased chemotherapeutic resistance. Furthermore, the expression of the stem cell-related genes, *SOX2, OCT4*, and *NANOG* are also upregulated in both the spheroid core and periphery populations compared to the 2D monolayer. For the 3D diffuse condition, *SOX2* showed a significant upregulation in expression compared to the 2D monolayer (48-fold; p < 0.001) while *NANOG* exhibited a modest, but not significant increase and *OCT4* showed no change in expression (Fold changes relative to the 2D monolayer are also shown in Supplementary Table 1).

### Chemotherapeutic Intervention Effect on CSC-related Genes

**Figure 4:**
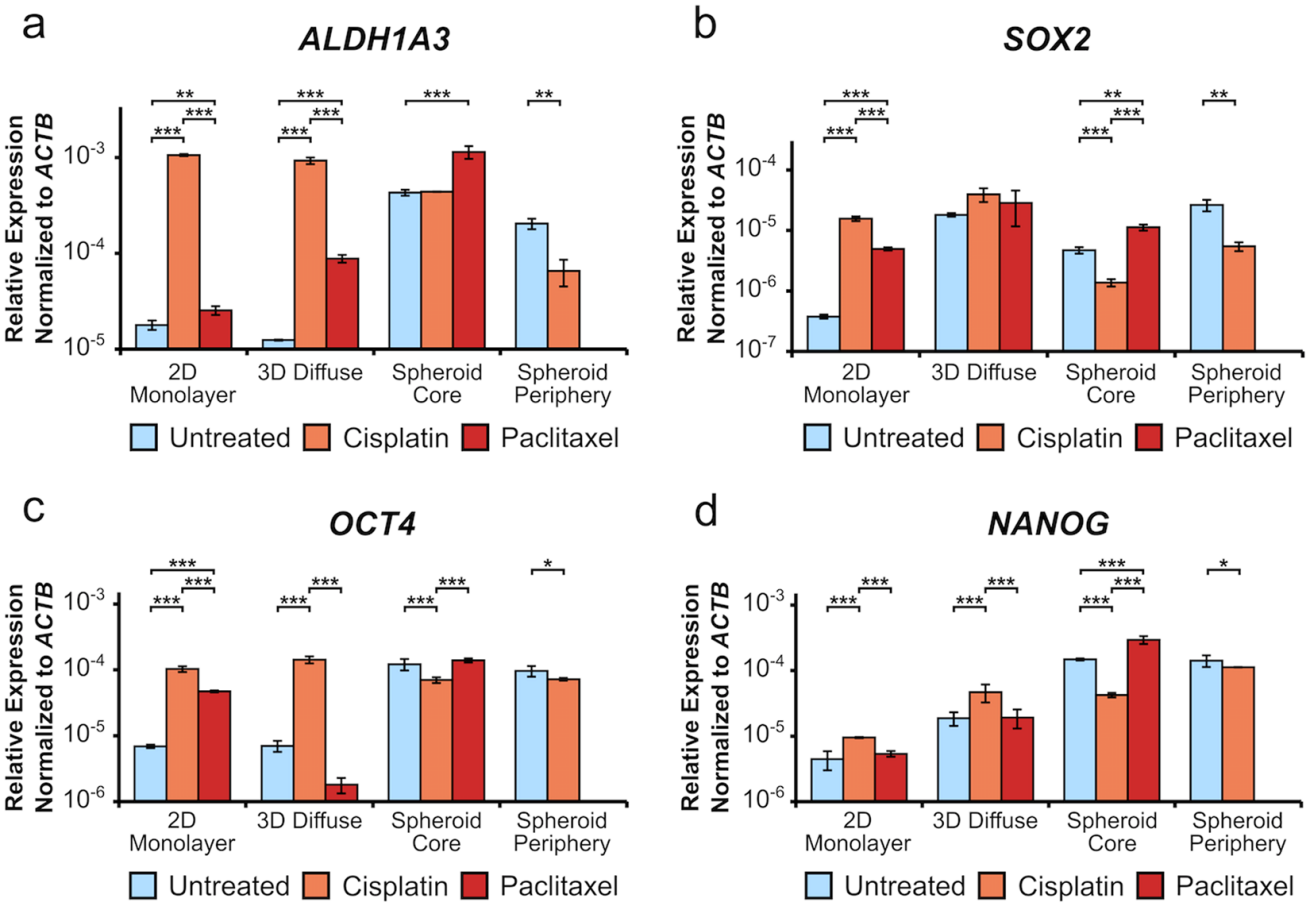
Paclitaxel treatment enriches for CSC markers, but cisplatin is context-dependent in MDA-MB-231 *in vitro* models. The gene expression of (**a**) *ALDH1A3*, (**b**) *SOX2*, (**c**) *OCT4*, and (**d**) *NANOG* following treatment with either cisplatin (orange) or paclitaxel (red) compared to untreated conditions (blue). All genes were analyzed in triplicate and the results are shown as mean ± SD. *P* values were determined using ANOVA with Tukey-Kramer post hoc analysis (*P < 0.05, **P < 0.01, ***P < 0.001). Note: paclitaxel treatment in the embedded 3D spheroid model effectively eradicated the periphery population.

Molecular confirmation of CSC content by assessing *ALDH1A3, SOX2, OCT4, and NANOG* expression, via the high sensitivity of the TaqMan assay, was subsequently obtained in each of our *in vitro* MDA-MB-231 models following treatment with either paclitaxel or cisplatin. Treatment with paclitaxel led to a statistically significant increase in *ALDH1A3* expression across all *in vitro* models compared to the corresponding untreated conditions (Fig. 4a): fold-changes of 1.42 ± 0.15; 7.10 ± 0.65; and 2.67 ± 0.40 (mean ± SD) were noted in the 2D monolayer, 3D diffuse, and 3D embedded spheroid core conditions, respectively. Therefore, the results suggest that paclitaxel treatments do enrich for cells expressing *ALDH1A3* although the extent of CSC present is significantly increased in the setting of a more clinically relevant 3D spheroid structure. There were increases in *SOX2* expression following paclitaxel exposure in all *in vitro* models with fold-changes of 13.11 ± 0.81, 1.72 ± 0.93, and 2.39 ± 0.27 (mean ± SD) in the 2D monolayer, 3D diffuse, and 3D spheroid core conditions, respectively (Fig. 4b); again supporting the observation by us and others^22, 33^ that paclitaxel treatment enriches for a stem-like subpopulation. Interestingly, treatment with the DNA intercalating agent cisplatin significantly increased the expression of *ALDH1A3*, *SOX2, OCT4*, and *NANOG* within the 2D monolayer and 3D diffuse systems, but did not increase these genes within the core or periphery populations (Fig. 4a,b). Fold-changes for each treatment condition with respect to the untreated control in each *in vitro* model are shown in Supplementary Table 2.

### Assessing the Effect of Chemotherapeutic Intervention on CSC-related Genes in the Luminal Breast Cancer Cell Line MCF7

**Figure 5:**
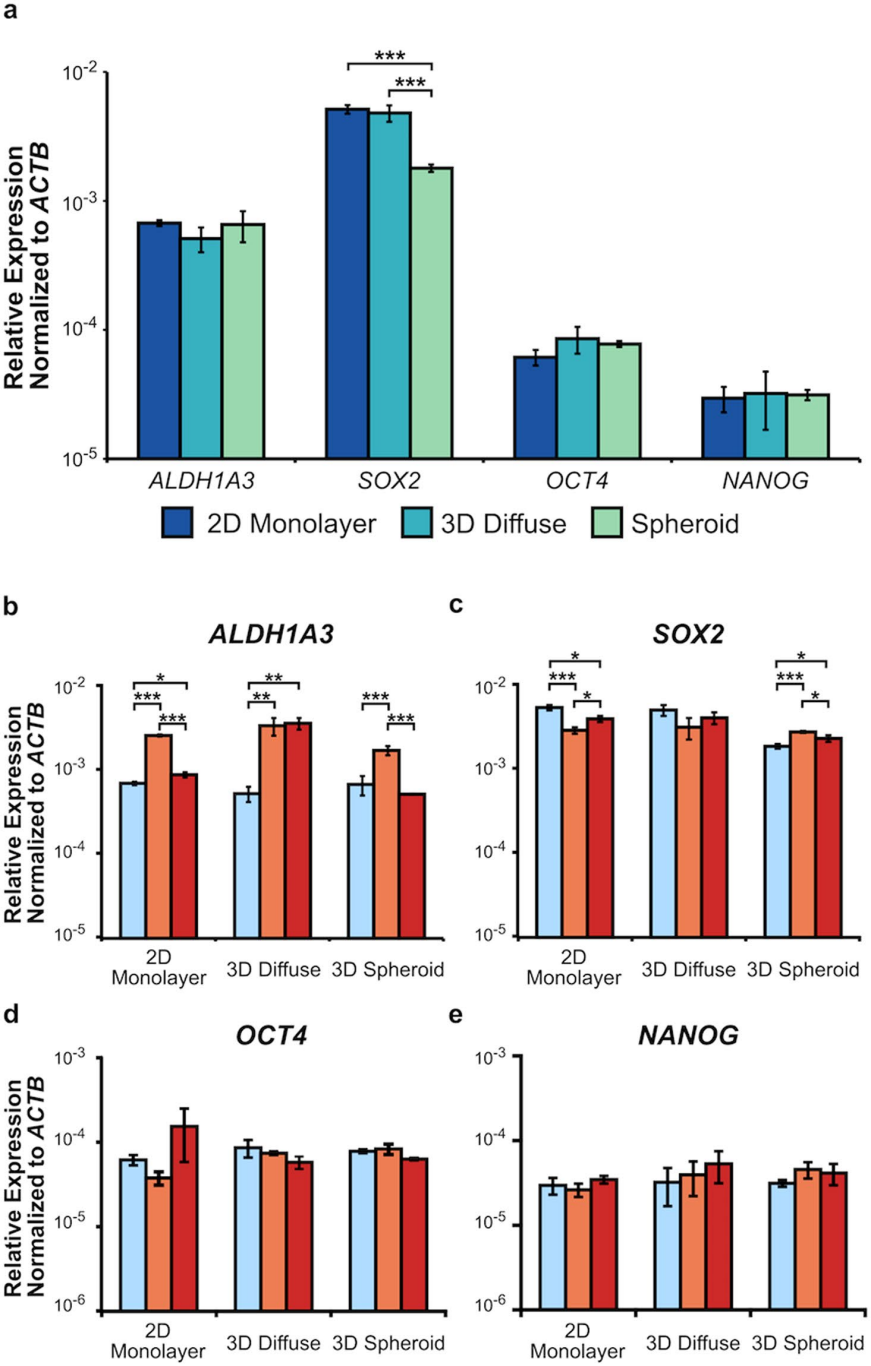
Expression of *ALDH1A3* in the luminal breast cancer cell line MCF7 is context-dependent following therapeutic assault. (**a**) RT-qPCR analysis of CSC-related genes in the untreated *in vitro* models for MCF7. The gene expression of (**b**) *ALDH1A3*, (**c**) *SOX2*, (**d**) *OCT4*, and (**e**) *NANOG* following treatment with either cisplatin (orange) or paclitaxel (red) compared to untreated controls (blue). All genes were analyzed in triplicate and the results are shown as mean ± SD. *P* values were determined using ANOVA with Tukey-Kramer post hoc analysis (*P < 0.05, **P < 0.01, ***P < 0.001).

To further investigate the relationship between our *in vitro* models and CSC-related gene expression, the expression of *ALDH1A3*, *SOX2, OCT4*, and *NANOG* was measured for the luminal breast cancer cell line MCF7 via the TaqMan assay (Fig. 5a). Here, MCF7 cells were found to have equivalent CSC-related gene expression independent of *in vitro* model with the exception of *SOX2*, which showed a fold-change of 0.35 ± 0.02 in the spheroid model as compared to the 2D monolayer (p < 0.001). It should be noted that the MCF7 spheroids did not exhibit invasion into the surrounding collagen and therefore did not possess a periphery population (Supplementary Fig. 4). As a result, the gene expression of the entire spheroid was assessed and labeled as ‘3D Spheroid’.

In addition, the CSC-related gene expression response to therapeutic assault by paclitaxel and cisplatin was evaluated for MCF7 within each of our *in vitro* models. Cisplatin treatment led to an increase in *ALDH1A3* expression across all *in vitro* models: 2D monolayer (3.67 ± 0.13; p < 0.001), 3D diffuse (6.32 ± 1.56; p < 0.01), and 3D spheroid (2.52 ± 0.35; p < 0.001) (Fig. 5b). However, cisplatin treatment decreased the expression of *SOX2* in the 2D monolayer (0.54 ± 0.05; p < 0.001) and increased its expression in the 3D spheroid (1.48 ± 0.04; p < 0.001) (Fig. 5c). Moreover, cisplatin treatment did not alter the expression of *OCT4* or *NANOG* in any of the *in vitro* models (Fig. 5d,e).

For paclitaxel, the expression of *ALDH1A3* increased in the 2D monolayer (1.27 ± 0.10; p < 0.05) and 3D diffuse (6.75 ± 1.14; p < 0.01) conditions, but remained unchanged in the 3D spheroid condition (Fig. 5b). The expression of *SOX2* decreased in the 2D monolayer (0.74 ± 0.06; p < 0.05) and increased in the 3D spheroid (1.24 ± 0.11; p < 0.05) conditions (Fig. 5c). However, paclitaxel treatment did not affect the expression of *OCT4* or *NANOG* (Fig. 5d,e). The fold-changes for each treatment condition with respect to the untreated control within each *in vitro* model are listed in Supplementary Table 3.

## Discussion

The 3D embedded spheroid model described herein recreates a phenotypic landscape, possessing an enriched and spatially-dependent CSC population, that reflects the phenotypic cellular heterogeneity found *in vivo*. Importantly, the results indicate that both the 2D monolayer and 3D diffusely embedded models are inadequate models to assess stemness and drug efficacy, findings that are supported by the clinical observation of chemoresistance despite *in vitro* susceptibility. While simply embedding single cells within 3D collagen matrices overcomes the limitation of forced cell polarization associated with 2D culture, it fails to impart important cell-cell interactions known to be intricately involved in promoting tumor progression^34–36^, and as such the 3D diffuse model did not perform better than the standard 2D monolayer assays.

The limitations of the various models are exemplified in our findings with MDA-MB-231 cells. The 2D monolayer model exhibited significantly greater susceptibility to both chemotherapeutic agents than either of the 3D embedded conditions, and this correlated with the lowest CSC content as measured by *ALDH1A3*. In the 3D diffuse model, cell viability following treatment with either paclitaxel or cisplatin was two-fold higher than the 2D monolayer and is similar to the cell viabilities found in the peripheral population from the 3D embedded spheroid model. The apparent difference in collagen invasion after treatment with paclitaxel and cisplatin has previously been observed in a colon cancer transwell migration assay^37^, and likely reflects paclitaxel’s mechanism of action—stabilization of the cytoskeleton microtubules. The observed differences in chemoresistance, viability, and CSC content are primarily due to the local 3D environment, given that the only difference between the models is the tumor architectures. If correct, this implies that the different tumor phenotypes, including chemoresistance, are malleable and more dependent on cell-cell interactions than previously appreciated.

Further analysis of the MDA-MB-231 3D embedded spheroid model reveals that the tumor response is not homogeneous throughout the tumor as drug efficacy and resistance are spatially-dependent, with the core cells exhibiting a more robust response compared to peripheral tumor cells. We hypothesize that the spatial-dependence of the 3D embedded spheroid response to drug treatment is a consequence of an increased CSC subpopulation present within the spheroid core cells compared to the cells in the spheroid periphery.

To address the potential chemoresistive role of the CSCs, we first determined the presence of CSCs in the various MDA-MB-231 2D and 3D models using markers of ALDH activity and the mammosphere assay. Results from the ALDEFLUOR assay, which identifies CSCs based on their ALDH activity, show an enriched ALDH + population within the embedded spheroid model compared to the 3D diffuse and 2D monolayer models. Further analysis reveals an ALDH + spatial-dependence with the spheroid core possessing a two-fold increase in ALDH + cells compared to the periphery population. Importantly, the mammosphere assay independently corroborates these findings with an enriched CSC subpopulation within the spheroid core (Fig. 3c). Subsequent gene expression analysis of *ALDH1A3*, an isoform of ALDH detectable by the ALDEFLUOR assay, accurately reflects the ALDEFLUOR results (Fig. 3d). Additionally, expression of the stem cell-related genes, *SOX2, OCT4*, and *NANOG* are upregulated in all spheroid conditions. Importantly, *SOX2* is a key transcription factor involved in embryonic stem cell self-renewal that is correlated with poor patient prognosis in breast cancer^31, 32, 38^.

Interestingly, when we investigated the expression of CSC-related genes in the luminal breast cancer cell line MCF7 without drug treatment, the expression of *ALDH1A3, OCT4*, and *NANOG* did not vary as a function of *in vitro* model (Fig. 5a). This result contradicts the enrichment of these genes seen in the MDA-MB-231 cells when cultured as 3D spheroids, and suggests that the CSC content within the MCF7 cell line is less sensitive to changes in tumor architecture, at least within our models. The results with the MCF7 cells are also in contrast to the report describing the expression of *SOX2* and *OCT4* increasing 2-fold when cultured within 3D collagen gels^39^. However, the collagen gel used in that study had a macroporous structure and was covalently crosslinked with 1-ethyl-3-(3-dimethyl aminopropyl) carbodiimide (EDC). The collagen gels used in these two studies possess different physical and mechanical properties, and, thus, these results suggest that CSC content can be altered or, potentially, controlled via use of a specific collagen gel composition - a focus of a future study.

Treatment with paclitaxel significantly enriches for *ALDH1A3* in all MDA-MB-231 *in vitro* models (Fig. 4a), and in the MCF7 2D monolayer and 3D diffuse models (Fig. 5b). This enriched CSC population following chemotherapeutic intervention may be due to effective intervention by paclitaxel against the non-stem-like population. In addition to the role of ALDH as a promoter of stemness through altered retinoid signaling^40^, ALDH also acts as a scavenger of reactive oxygen species (ROS)^33^. This ROS scavenging role provides a mechanism by which CSCs can evade therapeutic intervention through suppression of ROS-induced apoptotic mechanisms. Notably, there is experimental evidence indicating that at least part of paclitaxel and cisplatin’s efficacy is due to the generation of ROS^41, 42^. Interestingly, the MCF7 spheroid model did not show enrichment for *ALDH1A3* following paclitaxel treatment, indicating that the CSC and non-CSC MCF7 subpopulations exhibit equivalent resistance to paclitaxel within the spheroid model. This observation may reflect the clinical evidence showing that bulk populations of luminal breast cancers are more resistant to paclitaxel than other breast cancer subtypes^43, 44^

Cisplatin treatment significantly increased *ALDH1A3* expression in the MDA-MB-231 2D monolayer and 3D diffuse conditions, but did not affect its expression within the core population cells and actually reduced *ALDH1A3* expression in the periphery cells of the embedded model. The expression of *SOX2, OCT4*, and *NANOG* showed similar decreases within these populations as well. These results indicate that the chemotherapeutic response of MDA-MB-231s to cisplatin is context-dependent and is affected by multicellular architecture. Moreover, considering cisplatin’s greater efficacy at inhibiting growth in the reseeding experiments compared to paclitaxel, this impaired growth is likely due to cisplatin’s improved ability to affect CSCs within the spheroid microenvironment. To explain the context-dependent cisplatin result in MDA-MB-231 cells, we hypothesize that the relative proliferation rates between CSCs and non-CSCs influence the outcome. In 2D culture, CSCs proliferate more slowly compared to their non-CSC counterparts^45–50^, and given that cisplatin affects faster proliferating cells more effectively than slower proliferating cells^20^, we suggest that for conditions in which there are differences in the proliferation rates between CSCs and non-CSCs, cisplatin treatment will enrich for the slower proliferating CSC subpopulation. Therefore, we measured the presence of the proliferation marker Ki67 within each of our *in vitro* models (Supplementary Fig. 5). For the embedded spheroid model, we cryosectioned the spheroid to better measure the Ki67 levels in the core population. Our Ki67 immunostaining results support this hypothesis as the two *in vitro* models with the greatest proliferation, 2D monolayer and 3D diffuse, also exhibit an enriched CSC subpopulation following cisplatin treatment. For the spheroid conditions, we hypothesize that the lower overall proliferation rate, which is due to the *in vivo*-like multicellular architecture, no longer confers a selective advantage to the CSC subpopulation. Importantly, context-dependent responses are also seen within the MCF7 results as the cisplatin-treated 3D spheroid exhibited a smaller increase in *ALDH1A3* expression as compared to the 2D monolayer and 3D diffuse conditions (Fig. 5b). These context-dependent results illustrate the importance of investigating drug efficacy within multiple microenvironments in order to produce clinically relevant responses.

As for the minimal change in expression of *SOX2, OCT4*, and *NANOG* following therapeutic treatment in the MCF7 cells, this may be due to MCF7 being a luminal breast cancer subtype. Ben-Porath *et al*. reported that overexpression of these genes is correlated with the triple-negative basal breast cancer subtype, such as MDA-MB-231s, rather than the luminal subtype^30^. However, *ALDH1A3* expression is still a valid marker for CSC content in MCF7 cells as ALDH + MCF7s are more tumorigenic than their ALDH- MCF7 counterparts^51^.

In summary, we describe a tumor model that recapitulates the cell-cell and cell-matrix interactions found within the native tumor and its microenvironment, and it enables us to better characterize the CSC population dynamics in response to chemotherapy as a function of multicellular architecture. The difference in drug response between the spheroid core and periphery cell populations in the MDA-MB-231 cells demonstrates the need to evaluate these two populations separately. Whole system measurements or imaging simply do not reveal their disparities. Additionally, CSCs are enriched within the spheroid core while cells in the periphery contained fewer CSCs. Treatment with paclitaxel increased the CSC content of the spheroid core cell population as shown by the upregulation of CSC-related markers and the mammosphere assay. These results also show that paclitaxel or cisplatin alone do not effectively combat tumor growth—consistent with many clinical outcomes— and the results are model dependent (2D vs 3D). Given the complexity of *in vivo* tumors, opportunities exist to develop tumor models that enable studies from mechanistic cell-cell or cell-matrix signaling to drug screening^52^. To this end, the 3D embedded spheroid model recreates these interactions and reflects the cellular and metabolic heterogeneity found within tumors including the role of cancer stem cells, which are linked to chemoresistance, recurrence, metastasis, and progression.

## Materials and Methods

### Cell Culture

The post-metastatic, triple-negative breast cancer cell line MDA-MD-231 and the post-metastatic, luminal breast cancer cell line MCF7 were purchased from the American Type Culture Collection (ATCC) and cultured prior to experiments on tissue-treated polystyrene. The MDA-MB-231 cells were cultured in Dulbecco’s Modified Eagle’s Medium (DMEM) supplemented with 10% FBS and 1% penicillin/streptomycin solution for all *in vitro* models. The MCF7 cells were cultured in Eagle’s minimum essential media (MEM) supplemented with 10% FBS and 1% glutamine/penicillin/streptomycin. All experiments were performed at 37 °C in a humidified atmosphere containing 5% CO_2_.

### *In Vitro* Tumor Models

For the 2D monolayer *in vitro* model, cells continued to be cultured on tissue-treated polystyrene as previously mentioned. In the single-cell 3D diffusely embedded model, cells were embedded within 4 mg/mL collagen gels at a seeding density of 10^5^ cells/mL^53^. For the embedded spheroid model, spheroids composed of 10^4^ cells were formed following our published procedure with the addition of 2.5% Matrigel and centrifugation at 1,000 g for 10 minutes, and then embedded in 4 mg/mL collagen gels^17, 54^. In both 3D collagen models, cells/spheroids were transferred into unpolymerized collagen following our previously established procedure^17^. High Concentration Rat Tail Type I collagen (BD Biosciences) was combined 1:1 with a buffer solution (100 mM HEPES in 2x PBS, pH 7.3). This mixture was further diluted in PBS to the experimental collagen concentration of 4 mg/mL. The embedded cell/spheroid collagen mixture then self-polymerized into a gel after 1 h at 37 °C

### Chemotherapy Regimen

The treatment regimen for all models began 24 hours after initial seeding and consisted of 72 hours of drug exposure—10 ng/mL for paclitaxel (Indena) and 1.5 µg/mL for cisplatin (Sigma-Aldrich)—followed by removal of drug and an additional 72 hours of culture. Paclitaxel Oregon Green® 488 Conjugate (Thermo Fisher Scientific) was used for treatment in the same manner as paclitaxel. All viability data is presented with standard deviations.

### Extraction of Cells from *In Vitro* Models

Upon completion of each experiment, cells in the 2D monolayer were harvested using 0.05% trypsin and 0.02% EDTA (Sigma-Aldrich). For the 3D collagen models, Collagenase Type I (Invitrogen) was used at a concentration of 1.5 mg/mL in 1x PBS to dissociate the collagen gels. Moreover, for the embedded spheroid condition, collagenase treatment was used to isolate the periphery population from the intact spheroid core. To disaggregate the intact core, an additional treatment of 0.05% trypsin and 0.02% EDTA was performed.

### Viability Measurements

After treatment of models with chemotherapeutics as previously described, cells were extracted from both the 3D diffuse and embedded spheroid models. The resulting suspension of cells was seeded in a monolayer for 12 hours. The monolayer samples remain cultured during disaggregation of the three dimensional models. Cell viability was tested using a colorimetric MTS (3-(4,5-dimethylthiazol-2-yl)-5-(3- carboxymethoxyphenyl)-2-(4-sulfophenyl)-2H-tetrazolium) cell proliferation assay with absorbance read at 490 nm (Sigma). Cell viability in each well was calculated as a percentage of the untreated control of each model.

In order to test the ability of the spheroid core to grow after previous chemotherapeutic treatment, the spheroid was treated, and on the eighth day, the surrounding collagen and periphery cells were removed via treatment with collagenase at a concentration of 1.5 mg/mL in 1x PBS. The spheroid core was seeded into a 4 mg/mL collagen gel on oxoplates or tissue culture plates for imaging, and monitored for eight days without additional drug treatment. In order to gauge metabolic rate, oxoplates (PreSens Precision Sensing) measured the partial pressure of oxygen in the media. Measurements were conducted using a SpectraMax M5 plate reader (Molecular Devices) with ex/em of 540/590 and 540/650.

### ALDEFLUOR Assay

To quantify the CSC population within the aforementioned *in vitro* models, the ALDEFLUOR Stem Cell Identification and Isolation Kit (Stem Cell Technologies) was used in conjunction with a BD LSRFortessa (BD Biosciences) flow cytometer. Briefly, cells were incubated in ALDEFLUOR assay buffer containing ALDH substrate (1 µM) at a concentration of 5 × 10^5^ cells per mL for 30 minutes at 37 °C. For each experiment, a corresponding sample of cells was stained under identical conditions with 50 mM diethylaminobenzaldehyde (DEAB), a specific ALDH inhibitor, as a negative control. Propidium iodide (Abcam) was used to identify viable cells and sorting gates were established based on the DEAB-treated negative control.

### Mammosphere Assay

The mammosphere assay was performed by culturing cells isolated from the *in vitro* models on 24-well ultra-low adherence plates (Corning) in mammosphere growth media; which is composed of serum-free mammary epithelial growth medium (MEGM) bullet kit (Lonza), without addition of pituitary extract, and supplemented with B27 minus Vitamin A (Invitrogen), 20 ng/mL EGF and 20 ng/mL bFGF (BD Biosciences), 4 µg/mL heparin (Stem Cell Technologies). Cells were seeded at 1 × 10^3^ cells per well and half volumes of media were added every 3 days. After 10 days, brightfield images were acquired. Mammospheres were identified as multicellular aggregates larger than 70 µm in diameter and counted using ImageJ and a custom-written macro script (see SI).

### Gene Expression Analysis

Cell lysis and initial RNA extraction were performed using TRIzol Reagent (ThermoFisher) following manufacturer-recommended procedures. For the spheroid models, 18 spheroids were pooled for each replicate. After phase separation, RNA extraction was continued using an RNeasy Mini Kit (QIAGEN) with on-column DNase I treatment (QIAGEN), and RNA-to-cDNA was executed using the TaqMan High-Capacity RNA-to-cDNA Kit (Applied Biosystems). Gene expression was then assessed using the TaqMan Gene Expression Assay (Applied Biosystems) and a StepOnePlus RT-qPCR System (ThermoFisher). Details regarding TaqMan probes are listed in Supplementary Table 2. Data is presented with standard deviations.

### Microscopy

Images were acquired on a DMI600B microscope (Leica) with an ImagEM EM-CCD Camera (Hamamatsu Photonics, Hamamatsu, Japan) in a spinning disc confocal setup (Yokogawa). Imaging was done using Micro-Manager 1.4 Software (http://www.micro-manager.org). Assembling of frames into a single image was performed in ImageJ.

## Electronic supplementary material

Supplementary Information
