# Supplementary material for: Breast Cancer Spheroids Reveal a Differential Cancer Stem Cell Response to Chemotherapeutic Treatment
Source: Sci Rep. 2017 Sep 4;7:10382. doi: 10.1038/s41598-017-10863-4 (PMC5583341; doi:10.1038/s41598-017-10863-4)
Supplement: Supplementary file 1 — Supplementary Information [file 41598_2017_10863_MOESM1_ESM.pdf]

SUPPLEMENTARY INFORMATION

Breast Cancer Spheroids Reveal a Differential Cancer Stem Cell Response to Chemotherapeutic Treatment

Daniel S. Reynolds[1‡], Kristie M. Tevis[1‡], William A. Blessing[3], Yolonda L. Colson[2], Muhammad H. Zaman[1,4*], and Mark W. Grinstaff[1,3,5*]

1. Department of Biomedical Engineering, Boston University, Boston, MA 02215. 2. Division of Thoracic Surgery, Department of Surgery, Brigham and Women's Hospital, Boston, MA 02215. 3. Department of Chemistry, Boston University, Boston, MA 02215. 4. Howard Hughes Medical Institute, Boston University. 5. Department of Medicine, Boston University School of Medicine, Boston, MA 02118.

‡ contributed equally to the work

***Corresponding Authors**
Mark W. Grinstaff, Ph.D.
Boston MA, 02215
Tel: (617) 358-3429
Fax: (617) 358-3186
Email: mgrin@bu.edu

Muhammad H. Zaman, Ph.D.
Boston, MA 02215
Tel: (617) 358-5881
Fax: (617) 353-6766
Email: zaman@bu.edu

Supplementary Figure 1

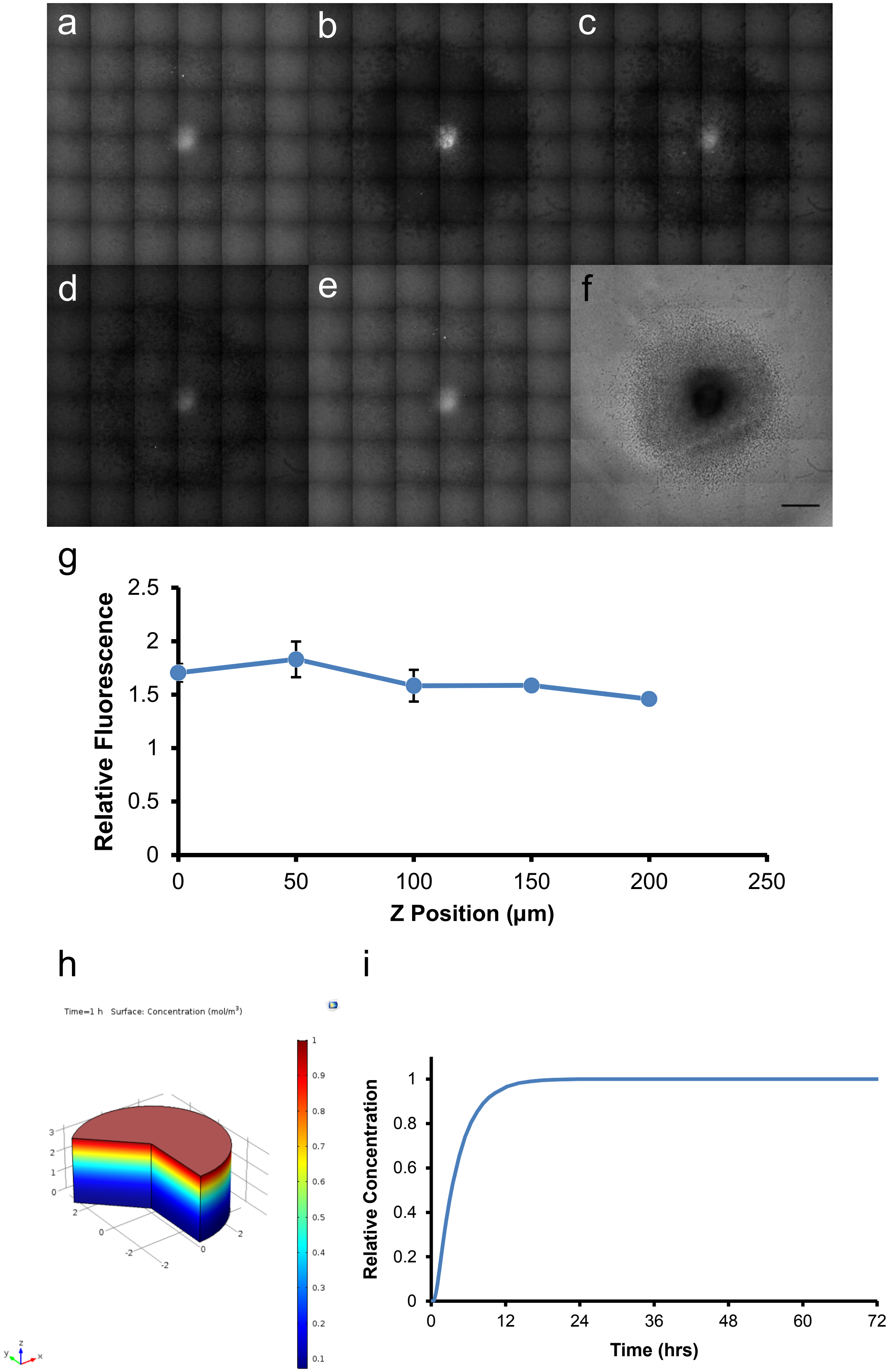

Supplementary Figure 2

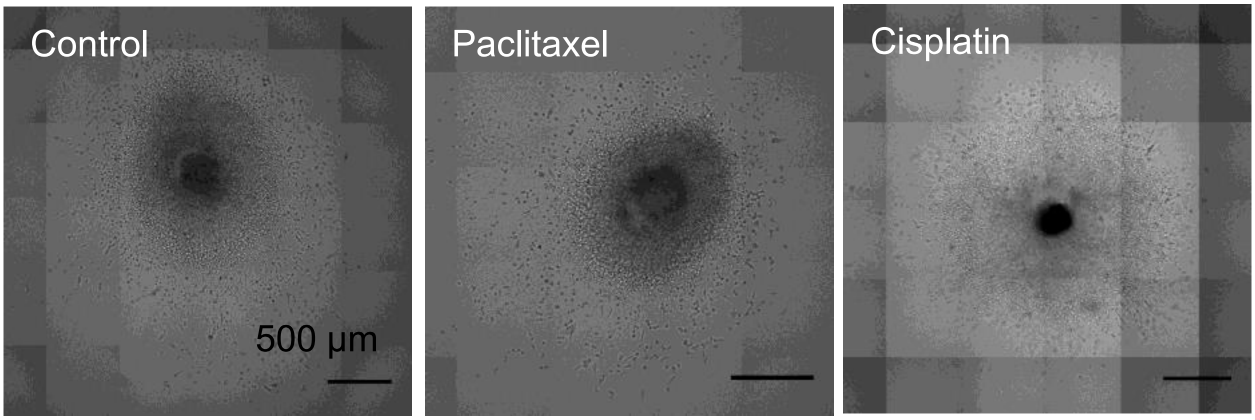

Supplementary Figure 3

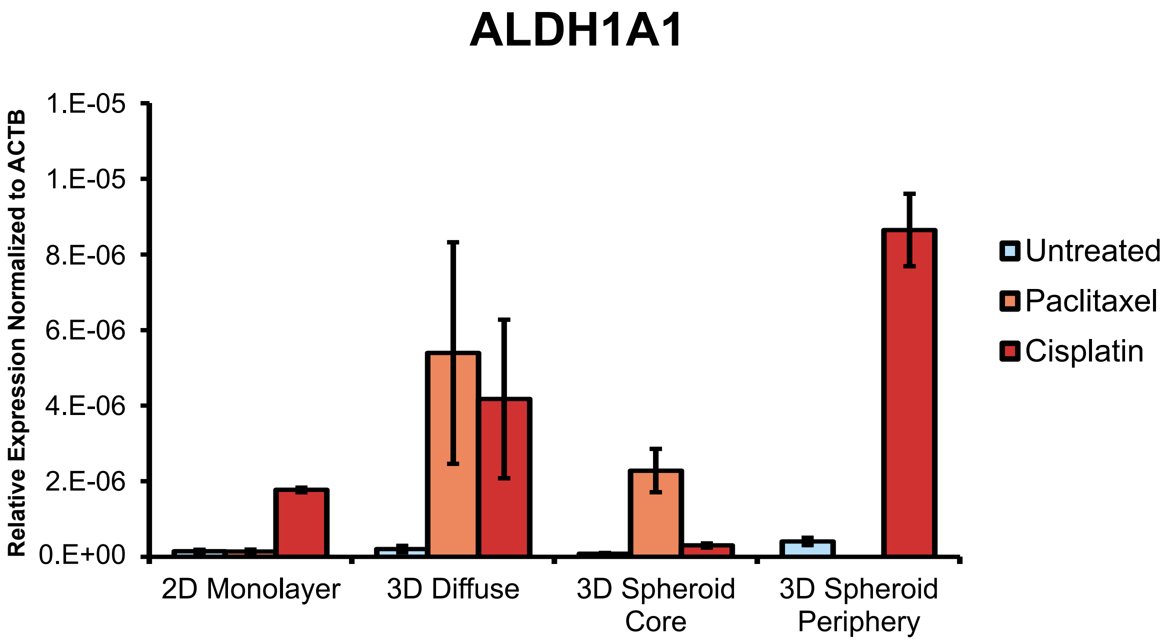

Supplementary Figure 4

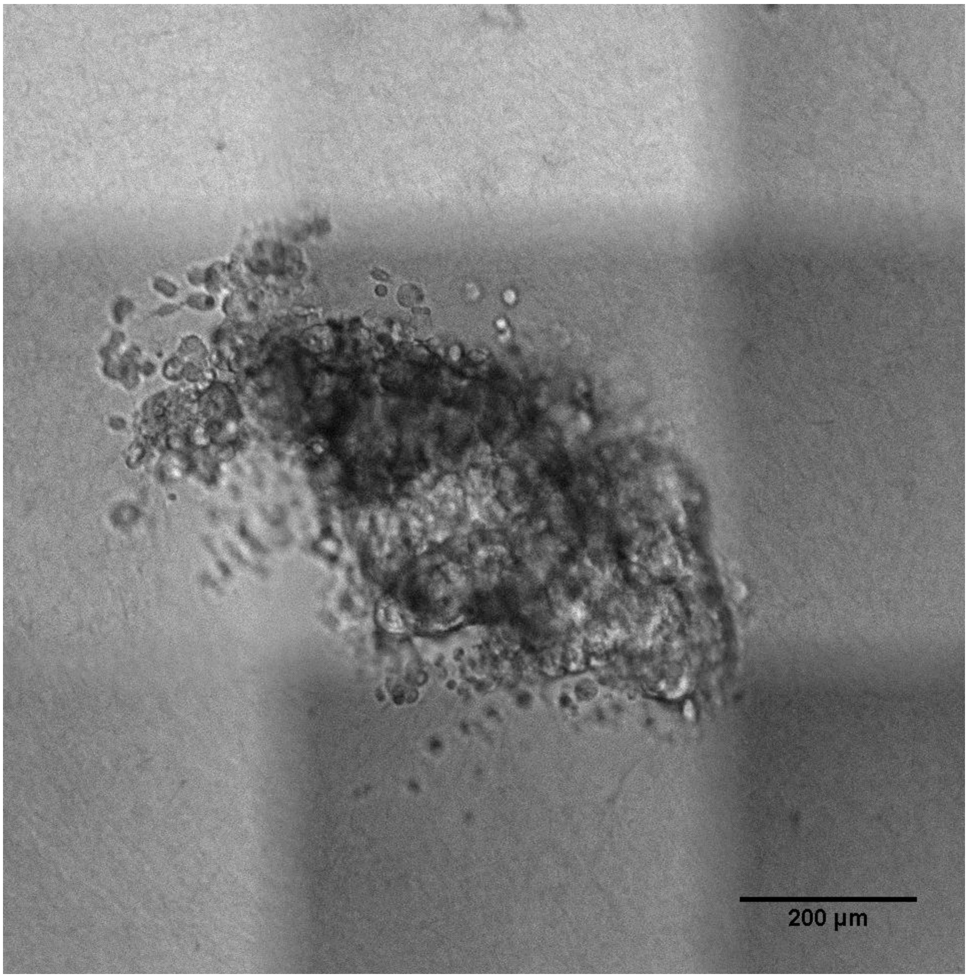

Supplementary Figure 5

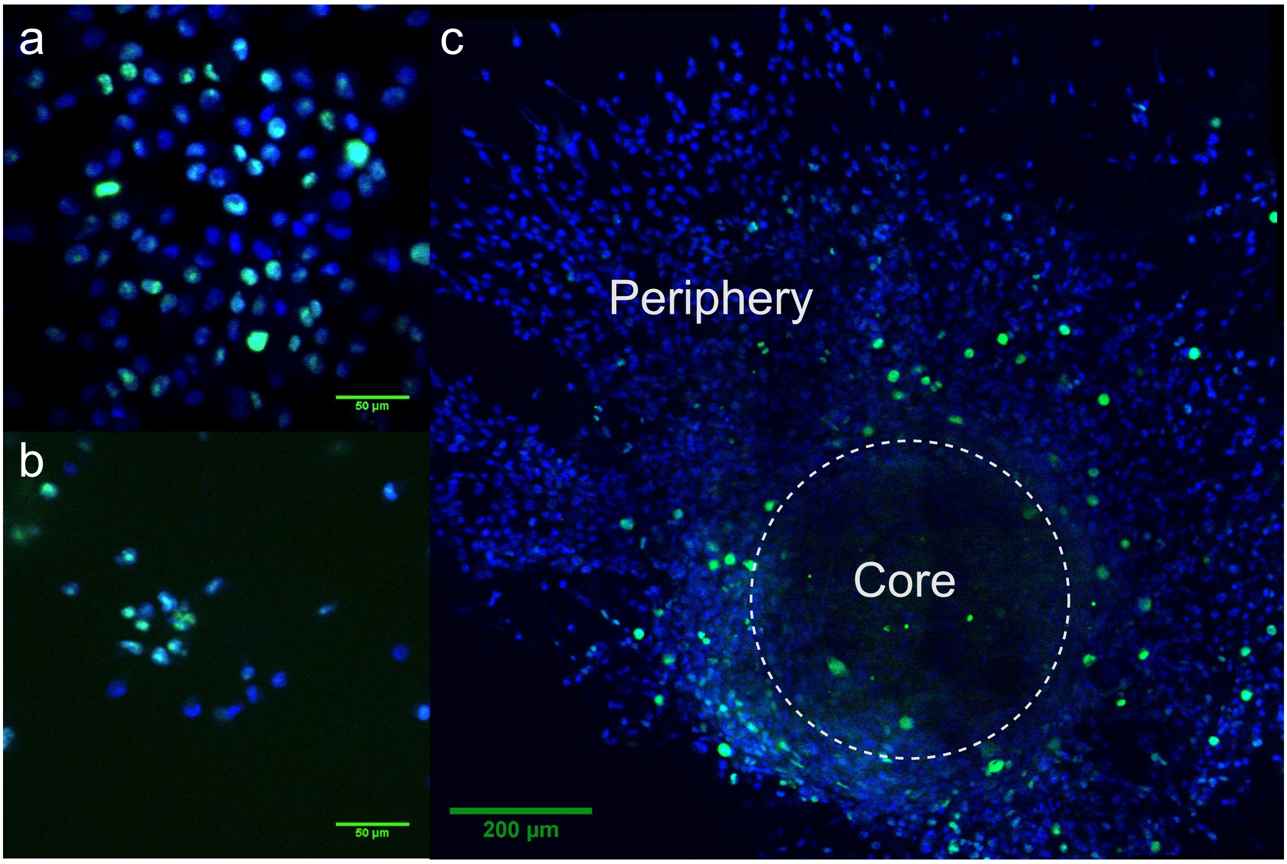

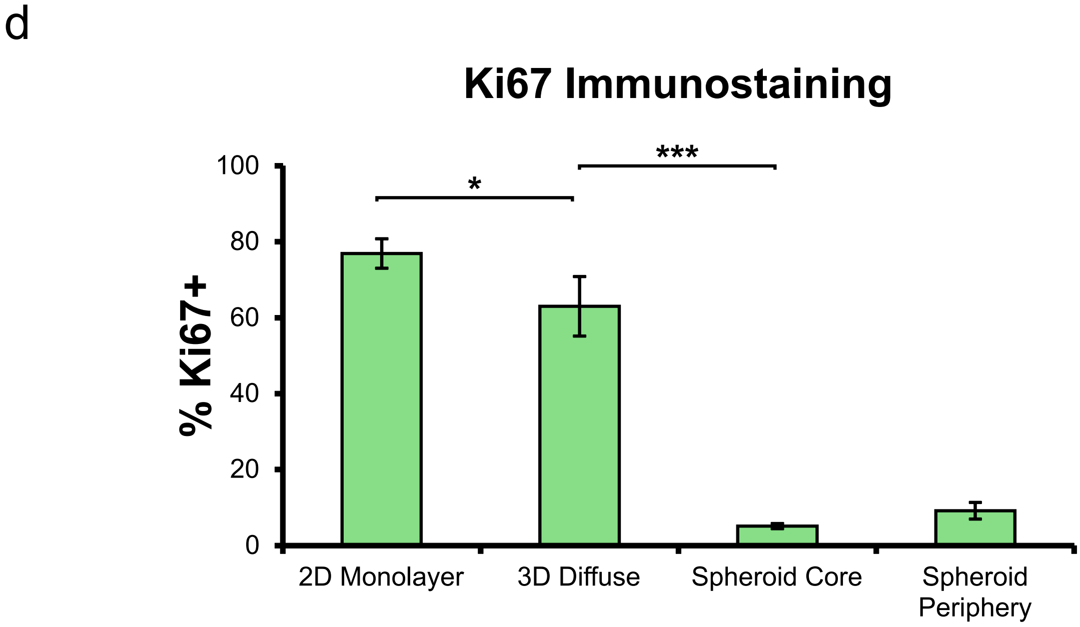

Supplementary Table 1

	2D Monolayer	3D Diffuse	Spheroid Core	Spheroid Periphery
ALDH1A3	1.00 ± 0.11	0.69 ± 0.04	23.90 ± 1.72	11.37 ± 1.32
SOX2	1.00 ± 0.13	48.20 ± 7.8	12.41 ± 1.45	69.41 ± 13.38
OCT4	1.00 ± 0.09	1.01 ± 0.20	17.70 ± 3.24	13.98 ± 2.71
NANOG	1.00 ± 0.29	4.21 ± 0.95	33.25 ± 2.62	31.80 ± 5.68

Supplementary Table 2

ALDH1A1	Untreated	Paclitaxel	Cisplatin
2D Monolayer	1.00±0.25	1.00±0.34	11.74±0.41
3D Diffuse	1.00±0.39	27.43±13.39	19.08±9.58
3D Spheroid Core	1.00±0.21	27.08±6.68	3.51±1.05
3D Spheroid Periphery	1.00±0.24	-	21.11±3.32

ALDH1A3	Untreated	Paclitaxel	Cisplatin
2D Monolayer	1.00±0.11	1.42±0.15	59.06±1.27
3D Diffuse	1.00±0.01	7.10±0.65	74.43±5.88
3D Spheroid Core	1.00±0.07	2.67±0.40	1.02±0.01
3D Spheroid Periphery	1.00±0.13	-	0.32±0.10

SOX2	Untreated	Paclitaxel	Cisplatin
2D Monolayer	1.00±0.08	13.11±0.81	41.40±3.53
3D Diffuse	1.00±0.07	1.72±0.93	2.18±0.55
3D Spheroid Core	1.00±0.13	2.39±0.27	0.29±0.07
3D Spheroid Periphery	1.00±0.22	-	0.21±0.03

OCT4	Untreated	Paclitaxel	Cisplatin
2D Monolayer	1.00±0.09	6.84± 0.45	14.96±1.67
3D Diffuse	1.00±0.19	0.26±0.09	20.36 ± 2.37
3D Spheroid Core	1.00±0.18	1.14±0.13	0.57±0.06
3D Spheroid Periphery	1.00±0.19	-	0.75±0.06

NANOG	Untreated	Paclitaxel	Cisplatin
2D Monolayer	1.00±0.29	1.21±0.32	2.14±0.27
3D Diffuse	1.00±0.23	1.03±0.34	2.50±0.67
3D Spheroid Core	1.00±0.08	1.99±0.28	0.29±0.06
3D Spheroid Periphery	1.00±0.20	-	0.80±0.08

Supplementary Table 3

ALDH1A3	Untreated	Paclitaxel	Cisplatin
2D Monolayer	1.00±0.05	1.27±0.10	3.67±0.13
3D Diffuse	1.00±0.22	6.75±1.14	6.32±1.56
3D Spheroid	1.00±0.27	0.77±0.01	2.52±0.35

SOX2	Untreated	Paclitaxel	Cisplatin
2D Monolayer	1.00±0.08	0.74±0.06	0.54±0.05
3D Diffuse	1.00±0.15	0.82±0.13	0.63±0.18
3D Spheroid	1.00±0.07	1.23±0.11	1.48±0.04

OCT4	Untreated	Paclitaxel	Cisplatin
2D Monolayer	1.00±0.14	2.49±1.55	0.61±0.11
3D Diffuse	1.00±0.23	0.67±0.11	0.86±0.04
3D Spheroid	1.00±0.05	0.81±0.03	1.07±0.15

NANOG	Untreated	Paclitaxel	Cisplatin
2D Monolayer	1.00±0.22	1.17±0.12	0.89±0.16
3D Diffuse	1.00±0.48	1.65±0.68	1.22±0.54
3D Spheroid	1.00±0.09	1.32±0.37	1.45±0.32

Supplementary Table 4

Gene	Assay ID
ALDH1A1	Hs00946916_m1
ALDH1A3	Hs00167476_m1
SOX2	Hs01053049_s1
POU5F1 (OCT4)	Hs00742896_s1
NANOG	Hs04399610_g1
ACTB	Hs01060665_g1

Supplementary Figure Legends:

Supplementary Figure 1. The MDA-MB-231 embedded spheroid model does not hinder transport of paclitaxel analog to cells. The embedded spheroid model was treated with Paclitaxel Oregon Green®, a fluorescent analog of paclitaxel, and imaged at five distinct z-positions: (**a**) 0 µm, (**b**) 50 µm, (**c**) 100 µm, (**d**) 150 µm, and (**e**) 200 µm. The embedded spheroid model imaged with DIC at 0 µm is shown in (**f**). The relative fluorescence of Paclitaxel Oregon Green® for the spheroid region, normalized to the surrounding background, was measured for each z-position (**g**). Results are shown as mean ± SD taken from three intensity profiles taken across each spheroid at random orientations. (**h**) The finite element model of small molecule chemotherapy drugs diffusing through our collagen gels is shown. Our experimental results revealed Irgacure 2959, which similar in size to cisplatin and paclitaxel, to have a collagen matrix diffusion coefficient on the order of $3.3 \pm 0.5 \times 10^{-10}$ m^2/s. (**i**) Incorporating these results into our finite element model enabled us to predict that the chemotherapy concentration within our collagen gels reaches equilibrium within 16 hours.

Supplementary Figure 2. Prior treatment of MDA-MB-231 embedded spheroid model does not prevent subsequent growth. Spheroid cores imaged growing in new collagen gels after prior treatment.

Supplementary Figure 3. Relative gene expression of *ALDH1A1* normalized to *ACTB* in MDA-MB-231. (a) *ALDH1A1* expression in the *in vitro* models for the

untreated (blue), paclitaxel (orange), and cisplatin (red) conditions. Error bars represent mean ± SD; n=3. *ALDH1A1* expression is significantly less than *ALDH1A3*, which is in agreement with recent literature showing that *ALDH1A3*, not *ALDH1A1*, is the ALDH isoform primarily responsible for ALDEFLUOR assay results and most closely correlated with the CSC phenotype.

Supplementary Figure 4. MCF7 spheroid morphology on Day 7. The untreated MCF7 3D spheroid model embedded within 4 mg/mL collagen gels on Day 7.

Supplementary Figure 5. Quantification of proliferation via immunofluorescent imaging of Ki67 within our MDA-MB-231 *in vitro* models. The presence of the proliferation marker Ki67 was measured across our *in vitro* models and representative images are shown: (**a**) 2D monolayer, (**b**) 3D diffuse, and (**c**) embedded spheroid. The boundary between the "Spheroid Core" and "Spheroid Periphery" populations was identified by visual inspection of cell morphology as cells remaining in the core exhibited elongated morphologies tangential to the spheroid surface. The percentage of Ki67+ cells were calculated using ImageJ's automated cell counting plug-in (**d**). The 2D monolayer had the greatest percentage of Ki67+ cells, and this was statistically significant from all other conditions. The spheroid periphery population had a modest increase in proliferation compared to the spheroid core, but it was still significantly lower than both the 2D monolayer and 3D diffuse conditions. The results are shown as

mean ± SD; n = 15 for the 2D monolayer and 3D diffuse conditions, n = 3 for the spheroid conditions. *P* value was determined using ANOVA with Tukey-Kramer post hoc analysis (*P<0.05, ***P<0.001).

Supplementary Table 1. Gene expression across MDA-MB-231 *in vitro* models normalized to the 2D monolayer. The fold-difference in gene expression is shown across *in vitro* models (normalized to 2D monolayer). Error bars represent mean ± SD; n=3.

Supplementary Table 2. MDA-MB-231 gene expression following paclitaxel or cisplatin treatments normalized to untreated conditions. The fold-change in gene expression is shown following chemotherapeutic intervention (normalized to the untreated condition within each *in vitro* model). Error bars represent mean ± SD; n=3.

Supplementary Table 3. MCF7 gene expression following paclitaxel or cisplatin treatments normalized to untreated conditions. The fold-change in gene expression is shown following chemotherapeutic intervention (normalized to the untreated condition within each *in vitro* model). Error bars represent mean ± SD; n=3.

Supplementary Table 4. List of TaqMan Probes used in this study.

Supplementary Methods:

COMSOL Diffusion Model:

We developed a finite element model using COMSOL Multiphysics to estimate the diffusion kinetics of cisplatin and paclitaxel within our 3D collagen gels[1]. We experimentally measured the diffusion coefficient of the small molecule Irgacure 2959 (Sigma), which is similar in size to cisplatin and paclitaxel, within our collagen gels using a technique adapted from a previously published protocol[1]. Briefly, Irgacure 2959 was loaded into our collagen gels at a concentration of 0.4 wt% and allowed to equilibrate overnight in 1x PBS containing 0.4 wt% of Irgacure 2959. Upon starting the experiment, the media was removed and replaced with fresh 1x PBS. Samples of media were taken over the course of a two-hour period, and due to Irgacure 2959's high absorbance in the UV spectrum, we were able to measure the diffusion kinetics of Irgacure 2959 into the surrounding media using a SpectraMax M5 Plate Reader. The diffusion coefficient of Irgacure 2959 within our collagen gels was then calculated using the semi-inifite slab approximation[2]. We incorporated this experimentally measured collagen diffusion coefficient into our finite element model, and used it to study the diffusion kinetics of cisplatin and paclitaxel.

Proliferation Measurements:

To quantify the proliferation rate within each *in vitro* model, we measured the presence of the proliferation marker Ki67 via immunofluorescence. For the 2D

monolayer condition, cells were fixed with 4% PFA for 15 minutes at room temperature and blocked with 1% BSA and 0.1% Triton X-100 for 1 hour at room temperature. Following blocking, samples were incubated with Ki67 primary antibody (PA5-16785, ThermoScientific (1:100 dilution)) overnight at 4°C. After incubation of the primary antibody, samples were incubated with an Alexa Fluor 488-conjugated secondary antibody (Invitrogen (1:500 dilution)) for 1 hour at room temperature. Nuclear staining was performed using the cyanine nucleic acid dye BOBO-3 Iodide (ThermoFisher) at a concentration of 10 µM and incubated at room temperature for 30 min. To improve the specificity of the nuclear stain, 0.2 mg/mL of RNase A (ThermoFisher) was added to the BOBO-3 staining solution. For the 3D diffuse condition, incubation times were increased two-fold to insure sufficient diffusion of the reagents throughout the collagen gels.

To assess the proliferation rate within the embedded spheroid model, we performed cryosectioning prior to immunostaining. Here, embedded spheroids were fixed with 4% PFA for 30 minutes at room temperature, embedded within OCT compound, and immediately immersed within liquid nitrogen. Cryosectioning was performed using a HM525 NX Cryostat (Thermo Fisher) and 300 µm-thick sections were taken. After cryosectioning, samples were thawed onto #0 coverslips. Immunofluorescent staining was performed in the same manner as the 3D diffuse conditions.

Code for Custom-written ImageJ Macro for Mammosphere Analysis:

```
setBatchMode(true);
for (i=1; i<=n; i++) {
		open("dir\\Pos_" + i + ".tif");
		run("Measure");
		run("Minimum...", "radius=3");
		run("Median...", "radius=5");
		setAutoThreshold("Intermodes");
		//run("Threshold...");
		setOption("BlackBackground", false);
		run("Convert to Mask");
		saveAs("Tiff", "dir\\Pos_" + i + "_binarized.tif");

		}
```

Supplementary References:

1. Chaudhuri, O. *et al.* Extracellular matrix stiffness and composition jointly regulate the induction of malignant phenotypes in mammary epithelium. *Nat. Mater.* **13,** 1–35 (2014).
2. Crank, J. *The mathematics of diffusion*. (Clarendon Press, 1979).
